# Strengthening midwifery and nursing interventions to reduce child mortality in South Asia: a policy and practice review

**DOI:** 10.7189/jogh.15.03041

**Published:** 2025-10-24

**Authors:** Lily Podder, Saikat Das, Kumari Dimple, Amit Agrawal, VR Vijay

**Affiliations:** 1 Nursing College, All India Institute of Medical Sciences, Bhopal, Saket Nagar, Bhopal, Madhya Pradesh, India; 2 Department of Radiation Oncology, All India Institute of Medical Sciences, Bhopal, Saket Nagar, Bhopal, Madhya Pradesh, India; 3 All India Institute of Medical Sciences, New Delhi, India; 4 Department of Neurosurgery, All India Institute of Medical Sciences, Bhopal, Saket Nagar, Bhopal, Madhya Pradesh, India; 5 College of Nursing, All India Institute of Medical Sciences, Raebareli, Uttar Pradesh, India

## Abstract

Child mortality continues to pose a major public health challenge in South Asia, where under-five and neonatal mortality remain high, especially in Afghanistan, Pakistan, and parts of India. Here, we synthesised evidence from 38 studies published between 2012 and 2024, examining predictors of mortality, barriers to effective nursing and midwifery interventions, and strategies to strengthen these roles. We identified four barrier domains: inadequate education and professional development, socio-cultural and gender constraints, infrastructure and resource shortages, and systemic policy challenges. Across several countries, over half of midwives lacked adequate pre-service training, more than 60% of facilities reported critical equipment shortages, and restrictive policies limited midwives’ autonomy. Despite these challenges, community-based interventions and targeted policy reforms demonstrated measurable improvements, including up to 49% increases in institutional deliveries and reductions in neonatal mortality by 2–3 per 1000 live births. Strengthening midwifery and nursing capacity through competency-based education, investment in rural health systems, and culturally sensitive community engagement – guided by World Health Organization and International Confederation of Midwives frameworks – represents a feasible pathway for reducing preventable child deaths and advancing progress toward Sustainable Development Goal 3 in South Asia.

Child mortality continues to be a major public health concern in South Asia, where under-five and neonatal mortality rates remain among the highest globally [1]. As of 2023, the region’s average under-five mortality rates stand at 35 per 1000 live births and neonatal mortality at 22 per 1000 – both exceeding the Sustainable Development Goal (SDG) rates of 25 and 12, respectively, to be achieved by 2030 [1]. While countries such as India have made significant steps in reducing under-five mortality from 93 in 1990 to 37 by 2020 [2], these improvements still differ across countries. For example, Nepal, Bangladesh, and Sri Lanka have seen substantial progress, with Sri Lanka achieving the lowest under-five mortality in the region, meeting SDG 3 targets [3–6], while Afghanistan, Pakistan, and parts of India continue to experience preventable child deaths [7–9]. These national estimates, however, are derived from diverse sources, such as household surveys (*e.g.* Demographic and Health Survey, National Family Health Survey (NFHS)) and civil registration systems, with varying levels of coverage and accuracy [10], limiting comparability and necessitating context-sensitive interpretation.

The disparity in child mortality outcomes across the region reflects not just economic differences, but also the varying strength of health systems and policy environments. For instance, Sri Lanka’s success is underpinned by a strong public health infrastructure, high female literacy, and centralised governance, which reinforce the effectiveness of midwifery and maternal health programmes [5,11,12]. In contrast, countries with fragmented governance, workforce shortages, or sociocultural resistance to institutional delivery face greater challenges in improving maternal and child health indicators [13]. Despite national flagship programmes, such as India’s Poshan Abhiyan and Swachh Bharat Mission, and Nepal’s Safe Motherhood Programme, the outcomes of these strategies often vary depending on implementation capacity, political continuity, and community engagement [14–16].

Addressing child mortality in this region requires more than clinical interventions; it demands comprehensive, systemic responses to long-standing barriers. These include uneven distribution of health workers, inadequate training infrastructure, low budgetary allocation to reproductive health, and weak regulatory frameworks [17]. Sociocultural factors (*e.g.* restrictive gender norms and community mistrust) further limit access to skilled care during childbirth and the postnatal period [18,19]. In response, countries have experimented with different strategic interventions, including the deployment of community health workers, midwifery education reforms, and the decentralisation of maternal health services [20,21]. However, the scale, quality, and sustainability of these reforms remain inconsistent.

Within these complexities, nurses and midwives have taken a leading role in advancing maternal and child health. Their roles extend beyond skilled birth attendance to include health education, antenatal counselling, postnatal monitoring, and community-level advocacy [22,23]. Evidence from South Asia suggests that midwifery-led models have improved newborn outcomes [24]; however, the effectiveness of such interventions depends heavily on broader structural supports. For example, qualitative studies from Afghanistan and Nepal highlight that outcomes improved in areas where midwives were locally recruited, received regular supervisory support, and were meaningfully integrated into community systems working in close coordination with local health committees, traditional birth attendants, and community leaders to strengthen trust, referral linkages, and service utilisation [25–27]. Conversely, in settings where they lacked autonomy, recognition, or supportive infrastructure, the impact was limited regardless of their training [28].

Despite the growing body of research on child mortality and its social determinants, the contribution of midwifery and nursing interventions remains underexplored in regional policy discourse [22,29]. Most national health systems still prioritise physician-led care, often overlooking the cost-effectiveness and social acceptability of nurse- and midwife-led approaches. In this viewpoint, we intended to fill the gap by systematically examining the predictors of child mortality in South Asia, assessing the role of midwifery and nursing interventions, identifying the barriers to their implementation, and proposing evidence-informed strategies for scaling and sustaining effective models. In doing so, we aimed to inform policymakers and practitioners seeking to strengthen health systems and accelerate progress toward SDG 3 across the region.

## APPROACH TO EVIDENCE SELECTION AND SYNTHESIS

We examined the predictors of child mortality and the role of midwifery and nursing interventions in South Asia, covering the period from January 2012 to July 2024. Although the study does not follow a systematic or scoping review design, we adopted a structured and transparent approach to ensure academic rigour. We conducted an extensive literature search in PubMed Central, Google Scholar, ScienceDirect, and ResearchGate.

We employed medical subject headings and keyword combinations such as (“Child Mortality” OR “Under-Five Mortality” OR “Neonatal Mortality”) AND (“Mortality Predictors” OR “Health Determinants”) AND (“Midwifery” OR “Nursing” OR “Nurses”) AND (“South Asia” OR individual country names) in our search strategy. We restricted the search to English-language publications with available full-texts, due to the lack of standard translation tools, and to maintain consistency in the screening process. Furthermore, we manually screened reference lists of selected articles to identify further eligible studies.

We included studies if they were conducted in South Asian countries (India, Pakistan, Bangladesh, Nepal, Sri Lanka, Bhutan, Maldives, or Afghanistan); provided empirical data (*i.e.* quantitative or qualitative findings derived from primary or secondary research) or policy-relevant evidence (*i.e.* analyses, reports, or reviews that inform or evaluate health policies and programmes related to child mortality); and examined the role of nurses and midwives in reducing mortality or described barriers to effective implementation. We excluded all articles that did not meet this inclusion criteria.

The authors (LP and KD) initially screened all titles and abstracts, followed by the full text of eligible studies. We did not formally assess their risk of bias or quality, and given the narrative nature of this viewpoint, we only prioritised peer-reviewed and evidence-based sources. We synthesised findings narratively and thematically, highlighting regional patterns, key predictors, and policy implications.

## KEY THEMES FROM THE EVIDENCE

### Characteristics of the included studies

**Figure 1:**
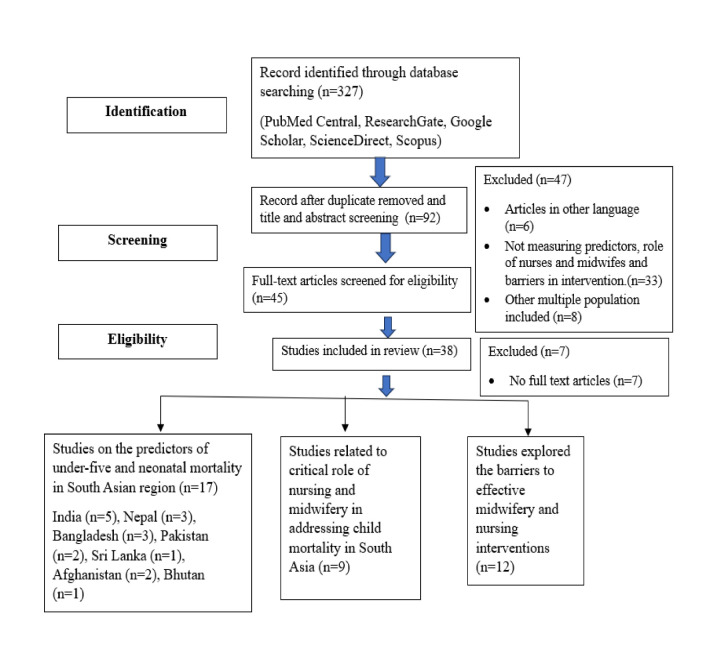
Flowchart showing search strategy and identification of studies.

We initially retrieved 327 articles from the databases. We then excluded 235 during title and abstract screening and 54 during full-text screening. Among the 38 included studies, 17 focussed on identifying predictors of under-five and neonatal mortality in South Asia, nine specifically examined midwifery and nursing interventions to reduce child mortality, and 12 addressed barriers to effective implementation of these interventions. Of the nine intervention-focussed studies, five were conducted in rural or remote regions, while four covered mixed urban and rural populations (**Figure 1**).

### Predictors of child mortality in South Asian countries

Seventeen studies focussed on predictors of under-five and neonatal mortality. These included five studies from India, three from Nepal, three from Bangladesh, two from Pakistan, two from Afghanistan, one from Sri Lanka, and one from Bhutan.

Due to heterogeneity in study designs, outcomes, and contexts, we employed a narrative thematic synthesis instead of a meta-analysis. We organised predictors into six mutually exclusive themes to minimise overlap: socioeconomic factors (structural and household determinants), maternal and child health characteristics (biological and care-related variables), geographic disparities, healthcare access, environmental exposures, and cultural/social influences. Neonatal-specific clinical factors were retained as a separate category to avoid redundancy (**Table 1**).

**Table 1:** Thematic classification of predictors of under-five and neonatal mortality in South Asia

Theme	Key predictors	Countries affected
Socioeconomic factors	Maternal, husband, household head education; maternal occupation; household wealth; access to electricity and sanitation; household size; place of residence; father occupation	India [30-34], Nepal [35,36], Bangladesh [37,38], Bhutan [39], Afghanistan [40], Pakistan [41,42], Sri-Lanka [43]
Geographic disparities	Geographical/residential region; distance to facility	India [30,33], Nepal [35,36,44], Pakistan [41], Sri-Lanka [43], Bhutan [39]
Maternal and child health	Maternal age at childbirth; birth interval; parity; child and maternal nutrition; breastfeeding status; birth weight; birth order; mode of delivery*	India [30-34], Nepal [35,44], Bangladesh [37,45], Afghanistan [40,46], Bhutan [39]
Healthcare access and utilisation	Use of antenatal/postnatal care; facility delivery; skilled birth attendance; mass media exposure; engagement with women’s groups; access to health information†	India [30,33], Bangladesh [45], Afghanistan [40]
Cultural and social factors	Early marriage; child’s gender; caste/religion	India [30,31], Nepal [35], Bangladesh [37], Afghanistan [46]
Neonatal-specific factors	Premature birth; low birth weight; neonatal infections (*e.g.* sepsis); birth asphyxia; shock	India [31,33,34], Nepal [35], Bangladesh [37], Bhutan [39], Afghanistan [46], Pakistan [42], Sri-Lanka [43]

*Birth weight, birth order, and mode delivery are also neonatal-specific factors.

†Engagement with women’s groups and access to health information are also neonatal-specific factors.

### The role of nursing and midwifery in addressing child mortality in South Asia

Nine studies examined nursing and midwifery interventions aimed at reducing child mortality in South Asia. Five were conducted in predominantly rural contexts (India, Nepal, Afghanistan, and Bangladesh), while four spanned mixed urban–rural settings (Pakistan, Sri Lanka, and multi-country analyses).

Global evidence supports the potential impact of these interventions. The 2014 Lancet Midwifery Series estimated that scaling up midwifery interventions could prevent 61% of maternal, foetal, and neonatal deaths in low human development index countries, rising to 83% when combined with family planning [47]. A Cochrane review found that midwife-led continuity of care reduces pre-term births and neonatal mortality [48]. Similarly, Nova and colleagues projected that universal midwife-delivered care could avert 67% of maternal deaths, 64% of neonatal deaths, and 65% of stillbirths by 2035, potentially saving 4.3 million lives annually [49].

In India, a nurse mentoring programme, implemented across eight districts of Karnataka from 2012 to 2015, was associated with a substantial reduction in neonatal mortality. These rates in nurse mentor–supported primary health centres fell from 29.4 to 9.3 per 1000 live births between baseline and endline surveys, with an adjusted hazard ratio of 0.23 (95% confidence intervals (CI) = 0.06–0.82, *P* = 0.02), while no significant change occurred in non-mentored facilities or home births [50]. Complementing this, a systematic review evaluating midwife-led units in India reported reduced neonatal intensive care unit-admission times, reflecting improved newborn health outcomes, which aligns with a broader systematic review of low- and middle-income countries, showing that midwife-led care reduces the odds of neonatal intensive care admission (odds ratio = 0.59; 95% CI = 0.44–0.75) [51,52].

Haththotuwa and colleagues reviewed the models of care responsible for Sri Lanka’s maternal newborn gains and emphasised the contribution of midwife-led primary care, routine home and community outreach, and strong referral mechanisms as proximate drivers of reduced maternal and neonatal deaths [53]. National monitoring data and World Health Organization (WHO)/United Nations Children’s Fund country analyses corroborate that near-universal skilled birth attendance and rapid postnatal follow-up services, primarily delivered by public health and hospital midwives, are associated with the country’s persistent low under-five and neonatal mortality rates [54].

Evidence from Pakistan reinforces these findings. For example, the Lady Health Worker programme, which incorporates trained community midwives into primary healthcare teams, has been associated with a 15% reduction in perinatal mortality and increased uptake of antenatal care and tetanus toxoid vaccination [55]. In rural Sindh, an intervention combining community midwives and female health visitors reduced neonatal mortality by 20%, with notable decreases in deaths from sepsis and birth asphyxia due to early detection and timely referral [56]. Afghanistan provides a striking example here; following rapid expansion of midwifery education programmes in the early 2000s, the proportion of births attended by skilled birth attendants more than tripled over the ensuing decade, coinciding with substantial reductions in perinatal mortality in multiple provinces [57].

Across these contexts, midwives and nurses have addressed the major causes of neonatal and under-five mortality, pre-term birth, low birth weight, sepsis, pneumonia, and diarrhoea through hygienic deliveries, antibiotic administration for neonatal infections, early breastfeeding promotion, and community-based management of childhood illnesses [58]. A systematic review of South Asian RCTs found that home-based newborn care delivered by community health workers significantly reduced neonatal and perinatal mortality, with the greatest benefits observed in high-mortality settings [59]. Large-scale studies in Bangladesh, India, and Pakistan further show that structured home visits by trained providers can reduce neonatal mortality by 30–61% and improve adherence to essential newborn care practices [60].

### Barriers to effective midwifery and nursing interventions

Across the included studies, 14 studies specifically reported barriers limiting the effectiveness of midwifery and nursing interventions in South Asia. We grouped them into four broad themes: educational and professional development constraints, socio-cultural and gender-related barriers, infrastructure and resource limitations, and systemic and policy-related challenges.

### Educational and professional development barriers

Across South Asia, foundational challenges hinder the establishment of midwifery as an autonomous profession. A regional report covering Afghanistan, Bangladesh, Bhutan, India, Nepal, and Pakistan found that none had legally recognised midwifery, only two had curricula aligned with International Confederation of Midwives (ICM) competency standards, and all faced inconsistent education, weak regulation, and fragile professional identity [61]. In India, 88% of nursing and midwifery education is private, midwifery remains subsumed within nursing, and career pathways are poorly defined, limiting skill development and recognition [62]. In Pakistan, qualitative evidence highlights a lack of structural ownership of the community midwives (CMW) programme, irregular stipends, no permanent service structure, and unclear regulatory frameworks for advancement [63,64]. In Bangladesh and Nepal, professional recognition is limited, policy support is weak, and professional associations remain underdeveloped despite expressed political will to align with global standards [65]. Additionally, limited research and evidence-based practice integration, inadequate faculty training and mentorship programmes, workforce migration and brain drain, and limited collaboration between midwifery education institutions and healthcare facilities further hinder professional growth and competency in the field [21,28,61,62,65–69].

### Sociocultural and gender-related barriers

Studies across South Asia show that entrenched cultural norms and gender biases limit midwifery effectiveness. Resistance to women in healthcare roles, inadequate accommodation, safety threats, gender discrimination, and preference for traditional birth attendants reduce uptake of professional services [21,66]. In Pakistan, community midwives face financial, transport, and security challenges [63]. In rural Chitwan, Nepal, distrust of young midwives leads families to prefer traditional attendants [70]. Evidence from Nepal, Bangladesh, and Pakistan highlights low community awareness, religious and cultural taboos, and minimal female leadership as barriers to care and policy reform [21,65,66,71,72]. Resistance from doctors and their associations undermines midwives’ autonomy and leadership in maternal health delivery [72].

### Infrastructure and resource constraints

In South Asia, nurses and midwives frequently encounter significant infrastructural barriers that compromise safe maternal and newborn care. A study of community midwives in rural Pakistan found that financial constraints, transportation difficulties, and security concerns were major obstacles limiting their ability to provide emergency obstetric and newborn care in their communities [63]. More broadly, low- and lower-middle-income settings, including those in South Asia, report frequent shortages of essential medical equipment, supplies, and medicines, along with underfunded infrastructure and weak referral systems, all of which severely constrain maternal healthcare delivery [73]. Poor digital connectivity and inadequate telehealth services restrict training opportunities and remote consultations, while the absence of designated spaces and support systems for independent midwifery practice reflects a broader neglect of infrastructure [21,28,64,74].

### Systemic and policy-related barriers

Weak policy integration and underfunding hinder midwifery and nursing interventions in South Asia. In India, midwifery remains subsumed under nursing, with no clear scope of practice, under-resourced regulatory bodies, and strong medical dominance, while a dedicated Midwifery Regulatory Act remains pending [62,75]. In Bangladesh, uncertainty persists over midwives’ authority to administer essential medications despite the development of a midwifery medicine list, which remained unsigned three years after approval, restricting clinical autonomy.[76] A recent scoping review across South-East Asia, including India and Nepal, further highlights the absence of legislation for community midwifery practice and inconsistent accreditation as major barriers to strengthening professional identity and service quality [21]. Furthermore, minimal stakeholder collaboration, resistance from physician associations, and the absence of clear legal provisions for independent nursing practice limit the potential of midwifery service expansion and leave many regions underserved [21,61,68].

### Strategic solutions to strengthen midwifery and nursing interventions for reducing child mortality in South Asia

Strengthening the midwifery and nursing workforce is crucial for reducing child mortality in South Asia. The WHO's State of the World's Midwifery Report and the Regional Strategic Directions for Strengthening Midwifery in the South-East Asia Region emphasise five key areas for investment: governance and regulation, education and training, workforce planning and management, practice and service delivery, and research and evidence. These elements provide a framework for addressing systemic barriers and enhancing the quality of midwifery-led healthcare [21,77].

Addressing the challenges in midwifery and nursing interventions in South Asia requires a comprehensive approach encompassing policy reforms, educational advancements, workforce improvements, and community engagement. The following solutions aim to strengthen the healthcare system, enhance midwifery and nursing education, and ensure better working conditions to improve maternal and newborn care outcomes (**Table 2**).

**Table 2:** Proposed solutions to strengthen midwifery and nursing interventions for reducing child mortality in South Asia

Proposed solution	Key strategies	Country-specific examples
Strengthening midwifery and nursing education	Implement CBE; enhance faculty, mentorship, continuous professional education	A review across Southeast Asia noted that CBE, with simulation and mentorship, significantly improved clinical competency and confidence in resource-limited settings [78].
Promoting awareness and gender equity in healthcare	Engage community and religious leaders to advocate midwifery; conduct gender-sensitisation campaigns	India’s Village Health and Sanitation Committees under the National Rural Health Mission have effectively included ANMs and ASHAs to mobilise community acceptance of female health providers [79].
Policy and regulatory reforms for midwifery integration	Establish national regulatory councils; align curricula with ICM; integrate midwifery into UHC policies	A regional review reported that only two of six South Asian countries have curricula aligned with ICM competencies, and none legally recognise midwifery as an autonomous profession [68].
Improving healthcare facilities and digital integration	Upgrade essential equipment; expand telehealth services for training and consultations	A telehealth pilot in rural Pakistan enhanced referral coordination and enabled remote consultations for maternal health, reducing delays in emergency care [80].
Enhancing career pathways and incentive-based retention policies	Provide rural service incentives, structured career paths, and professional recognition	Recognition of midwifery as a distinct profession, combined with targeted career development programmes, has been shown to improve retention and service quality in South Asia [21].

ANM – auxiliary nurse midwives, ASHA – accredited social health activists, CBE – competency-based education, ICM – International Confederation of Midwives

## POLICY IMPLICATIONS OF REGIONAL LESSONS

South Asia continues to face disproportionately high under-five and neonatal mortality, with India, Pakistan, and Bangladesh consistently among the top contributors worldwide [81]. This is driven by entrenched inequities in water and sanitation access, clean fuel use, and nutrition, alongside gaps in skilled birth attendance and quality perinatal care. NFHS-5 data reveal that in India, over 30% of households lack sanitation, 44% use unclean cooking fuel, and more than 30% of children are malnourished [82]. In contrast, smaller South Asian nations such as Sri Lanka, Bhutan, and the Maldives report nearly universal skilled birth attendance and antenatal/postnatal coverage, aided by robust primary healthcare systems [4,5,7,8,83–ss85]. These differences highlight the influence of health system design and governance structure on outcomes.

Policy experiences across the region reveal both gains and reversals. India’s Janani Suraksha Yojana boosted institutional delivery rates, but did not consistently lower neonatal mortality, partly because service quality, staffing, and supply chains lagged behind increased demand [86]. This underlines that financial incentives alone cannot substitute for system-wide strengthening. Evidence from the WHO midwifery framework and ICM standards suggests that trained, licensed, and regulated midwives can deliver up to 80% of essential maternal-newborn interventions, particularly when integrated with family planning.[49] Midwifery-led care is cost-effective, reduces unnecessary hospital referrals, and improves continuity [87,88], yet these benefits materialise only when implementation is supported by governance, regulation, and community trust.

Decentralised models such as Sri Lanka’s district health approach allow local adaptation, accountability, and sustained high coverage. In contrast, more centralised systems – *e.g.* Pakistan’s national midwifery programme – have struggled to embed services within communities, leading to underutilisation. Bangladesh’s inclusion of women’s groups in maternal health planning increased facility births by 18%, demonstrating how community participation improves service uptake [89]. The causal pathway is clear: community engagement enhances service utilisation, leading to reduced mortality, as demonstrated by a cluster-randomised review across South Asia showing that community mobilisation improved antenatal care uptake (risk ratio (RR) = 1.19; 95% CI = 1.06–1.33) and facility births (RR = 1.15; 95% CI = 1.11–1.20) [90]. Moreover, governance diversity shapes outcomes; for example, Kerala achieves near-universal institutional births (99.8%), in stark contrast to Uttar Pradesh (~67.8%), highlighting the importance of context-specific strategies rather than uniform national policies [91].

This review is limited by heterogeneity of study designs, uneven representation of countries, and the absence of robust causal evaluations for several government schemes. Findings should be interpreted with caution, as many reports emphasise programme outputs over measured mortality impacts. Future research should focus on longitudinal, mixed-methods evaluations of integrated maternal-newborn packages, comparative studies of decentralised health systems, cost-effectiveness analyses of midwifery workforce expansion, and deeper exploration of environmental determinants within local contexts. Ultimately, reducing child mortality in South Asia demands context-sensitive, governance-aligned, and community-grounded strategies that position midwives and nurses as the backbone of sustainable maternal-newborn care delivery.

## CONCLUDING REFLECTIONS

Reducing preventable child mortality in South Asia demands addressing interconnected barriers in education, professional development, sociocultural acceptance, infrastructure, and governance. While some countries show progress, persistent national and subnational disparities stem from uneven policy implementation, decentralised governance, and entrenched social norms. Evidence indicates that combining competency-based midwifery education, strong regulatory frameworks, rural infrastructure investment, and culturally sensitive community engagement can raise institutional delivery rates and lower neonatal mortality, but these gains are fragile without sustained investment, workforce retention, and recognition of midwifery as an autonomous profession.

Gaps remain in evaluating long-term sustainability, cost-effectiveness, and the impact of interdisciplinary collaboration. Most evidence derives from small-scale interventions, limiting generalisability. Future research should prioritise comparative, multi-country analyses to explain why similar policies succeed in some settings but falter in others, paying attention to policy reversals and implementation bottlenecks. Policymakers should integrate midwifery and nursing into broader health system reforms, align with WHO and ICM standards, and embed accountability mechanisms into national strategies. Without addressing these systemic and context-specific challenges, South Asia risks missing Sustainable Development Goal 3 targets despite ongoing investments in maternal and child health.
